# Early postoperative serum albumin levels as predictors of surgical outcomes in head and neck squamous cell carcinoma

**DOI:** 10.1016/j.bjorl.2021.03.004

**Published:** 2021-04-09

**Authors:** Tercio Guimarães Reis, Richard Alex Wesler Prudêncio da Silva, Eliane dos Santos Nascimento, José de Bessa, Márcio Campos Oliveira, Antônio Sérgio Fava, Carlos Neutzling Lehn

**Affiliations:** a Instituto de Assistência Médica ao Servidor Público Estadual, Pós-graduação em Ciências da Saúde, São Paulo, SP, Brazil; b Santa Casa de Misericórdia de Feira de Santana, Feira de Santana, BA, Brazil; c Hospital Otorrinos, Cuiabá, MT, Brazil; d Hospital Ipiranga, Departamento de Dietética, São Paulo, SP, Brazil; e Universidade Estadual de Feira de Santana (UEFS), Departamento de Saúde, Feira de Santana, BA, Brazil; f Hospital do Servidor Público Estadual Francisco Morato de Oliveira, Departamento de Cirurgia de Cabeça e Pescoço, São Paulo, SP, Brazil

**Keywords:** Malnutrition, Head and neck neoplasms, Nutrition assessment, Serum albumin, Postoperative complications

## Abstract

**Background:**

Malnutrition is a common issue in patients with head and neck squamous cell carcinoma and has a negative effect on surgical outcomes.

**Objective:**

We attempted to determine which malnutrition diagnostic variables can be used as predictors of postoperative complications in patients with head and neck squamous cell carcinoma.

**Methods:**

Forty-one patients undergoing surgery for head and neck squamous cell carcinoma were submitted to a prospective evaluation. Biochemical data, anthropometric measurements and evaluation of body composition were used in the nutritional analysis.

**Results:**

Twenty-two patients (53.6%) developed complications. Serum albumin measured on the first postoperative day was the only variable that significantly differed between groups. A cut-off value of 2.8 g/dL distinguished between patients with a complicated and uncomplicated postoperative course. Normalization of albumin levels occurred more frequently and more rapidly in the noncomplicated group.

**Conclusion:**

Serum albumin measured on the first postoperative day was the only variable that was a predicter of postoperative complications after major head and neck squamous cell carcinoma surgery.

## Highlights

• First-postoperative day (POD) serum albumin is a predictor of complications in head and neck surgery.
• First-POD albumin <2.8 g/dl increases the risk of postoperative complications.
• Non-complicated patients normalize serum albumin more often and five times more rapidly.

## Introduction

Malnutrition is a frequent issue in patients with head and neck squamous cell carcinoma (HNSCC), occurring in 20%–67% of cases.^1^, ^2^, ^3^ Alcohol abuse, tobacco smoking, poor oral hygiene and unhealthy eating habits all contribute to these patients having a certain degree of malnutrition prior to the onset of disease.^3^ HNSCC adds to the risk of malnutrition by directly disturbing the physiology of eating, thus leading to symptoms which affect mastication, impair food intake and preclude deglutition.^1^, ^2^, ^3^

Malnutrition, in turn, directly affects surgical outcomes, delaying wound healing and thus increasing the risk of infection, wound dehiscence, fistula formation and even carotid artery rupture.^4^ Undernourished patients therefore have increased mortality and morbidity and longer hospital stays, which directly lead to increased health-care costs.^5^

As there is no consensus regarding the assessment of malnutrition, multiple variables are used to detect it, many of which, however, do not appropriately reflect the patient’s nutritional status. In addition, despite having a lot of diagnostic tools of malnutrition in hospitalized patients, we do not know which nutritional parameters can predict surgical outcomes in HNSCC patients.

Although malnutrition is reported to occur frequently in HNSCC patients, little attention has been paid to its effect on surgical outcomes in this specific patient population. Considering the strong association between malnutrition and postoperative complications,^4^ the aim of this study is to determine which malnutrition diagnostic variables can be used as predictors of postoperative complications in patients with HNSCC.

## Methods

The study protocol was approved by the Research Ethics Committee, and all patients provided written informed consent, in adherence to the tenets of the local ethics committee (protocol number 0105/07).

This is a prospective cohort study involving patients admitted to the Department of Otorhinolaryngology/Head and Neck Surgery of the Hospital do Servidor Público Estadual de São Paulo Francisco Morato de Oliveira, from January 2008 to January 2009. Patients older than 18 years of age who were eligible to en bloc surgery (removal of the primary tumor and neck dissection) for the treatment of T2–T4^6^ HNSCC were included in the study. Patients were excluded if they refused to participate in the study or met one of the following criteria: younger than 18 years of age, preoperative use of nutritional supplements, prolonged use of corticosteroids, dyslipidemia, nephrotic syndrome, congestive heart failure, hepatic insufficiency or any other disease affecting the normal metabolism of hepatic proteins.

### Preoperative evaluation

Patients were admitted to the hospital 72 h before surgery and the following data were included in the study protocol form: age, sex, monthly household income,^7^ education level,^8^ primary tumor subsite, clinical stage,^6^ comorbidities, previous head and neck surgery or radiotherapy.

Nutritional analysis was performed 24 h before surgery by a nutritionist and consisted of anthropometric measurements and body composition assessment by bioelectrical impedance analysis (BIA).

### Anthropometric evaluation

A measuring tape and a scale (Welmy®) were used to measure height and weight. The body mass index (BMI) was obtained by dividing the weight in kilograms by the square of the height in meters.^9^

A Lange® skinfold caliper was used to measure the triceps skinfold (TSF) on the nondominant arm, and a measuring tape was used to determine the arm circumference (AC) and the calf circumference (CC). Measurements were performed in triplicate to the nearest 1-mm.^10^, ^11^

The following formula was utilized to calculate the percentage of ideal body weight (% IBW): (AW/IW) × 100, with “AW” being the actual weight and “IW”, the ideal weight. The 1983 Metropolitan Life Insurance Company tables were used to calculate the IW based on sex, height, age, and body frame size. The %IBW was used to classify patients as having mild (80%–90%), moderate (70%–79%) or severe (<69%) malnutrition.^12^

The following formula was used to calculate the percentage of weight change (%WC): (AW − UW)/UW × 100, where “AW” is the actual weight and “UW”, the usual weight. A %WC of <5% indicates a small weight loss, while 5%–10% suggests a potentially significant weight loss and >10%, a definitely significant one.^13^ Additional data specifying whether the weight loss was continuous or interspersed with weight recovery periods were also obtained.

### Body composition analysis

All patients underwent bioelectrical impedance analysis according to the National Institute of Health standards.^14^ A bioelectrical impedance analyzer (Biodynamics®, 310e, version 8.0) was used to determine resistance and reactance, which were then utilized to estimate total body water (TBW), body fat (BF) and bean Body mass (LBM).

### Nutritional support

Patients were given no nutritional support during the preoperative period. The Harris–Benedict equation^15^ (66,437 + (5,0033 × height [cm]) + (13,7516 × weight[kg]) − (6,7550 × age, to men and 655,0955 + (1,8496 × height [cm]) + (9,5634 × weight[kg]) − (4,6756 × age to women) was used to estimate individual basal energy expenditure (BEE) and total energy expenditure (TEE). All patients received postoperative nutritional support, which was enterally given.

### Biochemical evaluation

The following laboratory tests were performed: complete blood count, total serum protein and its fractions (colorimetric assay), total cholesterol and its fractions (enzymatic colorimetric assay) and absolute lymphocyte count (ALC), as determined by the following formula: (% lymphocytes × leukocytes)/100. An ALC of 1,200–2,000 cells/mm³ suggests mild depletion, while a result of 800–1,200 cell/mm³ indicates moderate depletion and <800 cells/mm³, severe depletion.^16^

## Surgery

Data regarding the type of surgery and neck dissection, the need for flap reconstruction, the occurrence of intraoperative complications and the transfusion of blood products were collected.

Povidone-iodine (PVP-I) was used for skin preparation before surgery and irrigation with warmed 0.9% normal saline was carried out prior to surgical wound closure.

Antibiotic prophylaxis consisted of ceftriaxone and clindamycin administered intravenously at the induction of anesthesia and continued for the following 48-h.

### Postoperative course

Patients were transferred to the intensive care unit in the immediate postoperative period. Enteral feeding was initiated 12–48 h after surgery. Serum albumin was measured on the first, third, fifth and seventh postoperative day (POD). Some patients required blood transfusion to keep hemoglobin (Hb) levels above 10 g/dL.

Clinical, biochemical and radiographic features were used to diagnose postoperative complications. The following “major complications”, as defined by Van Bokhorst-de van der Schueren et al., were looked for: surgical wound infection (spontaneous or surgical purulent drainage); flap necrosis (ischemic injury); oro- or pharyngocutaneous salivary fistula; anastomotic leakage; septic shock; respiratory failure requiring ventilatory assistance for longer than 12 h or return to mechanical ventilation after extubation; and death.^17^ The diagnosis of pneumonia was based on the Brazilian guidelines for the treatment of hospital-acquired and ventilator-associated pneumonia.^18^

Patients who underwent total laryngectomy underwent a videofluoroscopic evaluation of swallowing between the seventh and tenth POD; in all other cases, a test consisting of the oral administration of methylene blue diluted in water was used for the diagnosis of fistula.

All patients included in the study were followed until discharge from the hospital.

### Statistical analysis

Data were expressed as means ± standard deviation (SD), medians and interquartile ranges or absolute values and fractions. The Student *t*-test or Mann–Whitney test were used to compare continuous variables, while the Chi-Square test was used to compare categorical variables.

ROC (receiver operating characteristic) curves were used to assess the predictive properties of the variables under analysis. The area under the ROC curve determined the overall discriminating power of nutritional variables to predict postoperative complications; in addition, the cut-off values with the best predictive power were identified.

The log-rank test (Mantel–Cox test) was used to compare albumin normalization curves, while hazard ratios (HR) and their respective confidence intervals (CI) were used to estimate the magnitude of potential differences.

Logistic regression will be used to analyze the relationship between categorical dependent variables and independent variables.

All tests were two-sided, with *p*-values < 0.05 considered statistically significant. Commercially available statistics software GraphPad Prism (version 6.02 for Windows, San Diego, CA, USA) was used to perform statistical analysis.

## Results

Forty-one patients were included in the study, 36 men (87.8%) and 5 women (12.2%), with an overall mean age of 61.4 years. Additional sociodemographic data on the study population are shown in Table 1.

**Table 1:** Correlation between sociodemographic data, preoperative and postoperative clinical data and the risk of postoperative complications.

	Complicated (n = 22)	Noncomplicated (n = 19)	*p*-Value
**Age**	61.32 ± 10.77	61.57 ± 10.42	0.941
**Sex**			
Male	18 (82%)	18 (95%)	0.707
Female	4 (18%)	1 (5%)	
**Monthly income**^a^			
Up to 3 times the minimum wage	9 (41%)	8 (42%)	0.969
Between 3 and 7 times the minimum wage	10 (45.4%)	8 (42%)	
>7 times the minimum wage	3 (13.6%)	3 (16%)	
**Education level**^b^			
Illiterate	1 (4.5%)	0	0.531
Incomplete primary education	10 (45.5%)	8 (42.1%)	
Complete primary education	4 (18.2%)	6 (31.6%)	
Incomplete secondary education	1 (4.5%)	3 (15.8%)	
Complete secondary education	2 (9.1%)	0	
Higher education	4 (18.2%)	2 (10.5%)	
**Primary tumor subsite**			
Oral cavity	9 (40%)	6 (31.6%)	0.083
Oropharynx	6 (27.3%)	1 (5.2%)	
Hypopharynx	3 (13.6%)	2 (10.5%)	
Larynx	4 (18.2%)	10 (52.7%)	
**Clinical stage**			
II	2 (9.1%)	4 (10.5%)	0.533
III	7 (31.8%)	6 (31.6%)	
IV	13 (59.1%)	9 (47.4%)	
**T (tumor size)**			
T2	4 (18.2%)	7 (36.8%)	0.339
T3	11 (50%)	6 (31.6%)	
T4	7 (31.8%)	6 (31.6%)	
**Flap reconstruction**			
Yes	7 (31.8%)	4 (21.1%)	0.437
No	15 (68.2%)	15 (78.9%)	
**Blood transfusion**			
No	11 (50%)	13 (68.4%)	0.232
Yes	11 (50%)	6 (31.6%)	
**Initiation of feeding**			
Up to 12 h after surgery	4 (18.2%)	8 (42.1%)	0.247
12 to 24 h after surgery	14 (63.6%)	10 (52.6%)	
24 to 48 h after surgery	2 (9.1%)	0 (0)	
No data	2 (9.1%)	1 (5.3%)	
**Comorbidities**			
Yes	13 (59.1%)	13 (68.4%)	0.536
No	9 (40.9%)	6 (31.6%)	
**Previous radiotherapy**			
Yes	3 (13.6%)	1 (5.3%)	0.367
No	19 (86.4%)	18 (94.7%)	
**Previous surgery**			
Yes	5 (22.7%)	1 (5.3%)	0.114
No	17 (77.3%)	18 (94.7%)	

Data are expressed as mean ± standard deviation or number (%) of patients.

a Monthly minimum wage in 2008: R$450.00.^9^

b Law on Brazilian Education Guidelines and Bases (9,394/1996).^10^

All patients had a histopathologic diagnosis of HNSCC: 15 of the oral cavity (36.6%), 7 of the oropharynx (17%), 5 of the hypopharynx (12.2%) and 14 of the larynx (34.2%). Regarding clinical staging, most patients (22) had stage-IV HNSCC (53.7%).

Twenty-four patients (58.5%) underwent unilateral neck dissection, while 17 (41.5%) were submitted to a bilateral procedure. All subjects needed a postoperative tracheostomy. Only 26.8% of patients needed flap reconstruction: 4 of them with a microsurgical flap and 7 with a pectoralis major myocutaneous one (Table 1).

Of all included cases, 22 patients (53.6%; 95% CI 38.74%–67.95%) had postoperative complications, but no patient died during follow-up.

No demographic variable was significantly correlated with the occurrence of postoperative complications (Table 1). Regarding clinical features, there was no statistically significant difference between groups for clinical stage, presence of comorbidities, primary tumor subsite, previous surgery or radiotherapy, transfusion of blood products or the need for flap reconstruction (Table 1).

Of the 41 study patients, 35 were submitted to body composition evaluation by bioelectrical impedance analysis. There was no statistically significant difference between groups for total body water (*p* =  0.1341), body fat (*p* =  0.2051) or lean body mass (*p* =  0.2167) (Table 2).

**Table 2:** Correlation between preoperative anthropometric data, preoperative bioelectrical impedance analysis, preoperative biochemical and hematological data, and the risk of postoperative complications.

	Complicated (n = 22)	Noncomplicated (n = 19)	*p*-Value
**%IBW**^a^			
>90%	17 (77.3%)	12 (63.2%)	0.517
80%–89%	2 (9.1%)	4 (21.1%)	
No data	3 (13.6%)	3 (15.7%)	
**%WC**^b^			
None	6 (27.3%)	5 (26.3%)	0.316
≤5%	2 (9.1%)	6 (31.6%)	
5%–10%	9 (40.9%)	5 (26.3%)	
≥10%	5 (22.7%)	3 (15.8%)	
**Type of weight loss**			
None	6 (27.3%)	5 (26.3%)	0.547
Continuous	11 (50%)	12 (63.1%)	
Interspersed with weight recovery periods	5 (22.7%)	2 (10.6%)	
**Anthropometric data (cm)**			
Triceps skinfold	1.158 ± 0.48	1.200 ± 0.63	0.811
Arm circumference	26.93 ± 2.73	28.08 ± 3.82	0.269
Calf circumference	29.49 ± 5.70	32.59 ± 5.07	0.341
Body mass index (kg/m²)	23.19 ± 3.74	23.09 ± 3.96	0.934
**Bioelectrical impedance**			
% total body water	51.71 ± 7.85	54.94 ± 3.78	0.1341
% lean body mass	67.38 ± 8.44	70.69 ± 7.02	0.2167
% body fat	31.44 ± 7.89	28.19 ± 6.92	0.2051
**Absolute lymphocyte count**^c^			
Normal	11 (50%)	6 (31.6%)	0.339
1,200–2,000 mm^3^	7 (31.8%)	11 (57.9%)	
800–1,199 mm^3^	3 (13.6%)	2 (10.5%)	
<800 mm^3^	1 (4.6%)	0 (0)	
**Hematological data**			
Hemoglobin	13.88 ± 1.58	13.95 ± 1.66	0.891
Hematocrit	41.28 ± 4.62	41.38 ± 4.8	0.946
**Biochemical data**			
Total cholesterol	191.9 ± 44.4	182.9 ± 9.613	0.526
HDL	48.15 ± 14.9	48.45 ± 15.23	0.949
LDL	114.15 ± 39.57	112.31 ± 34.57	0.875
Total protein	6.98 ± 0.63	7.04 ± 0.61	0.759

Data are expressed as mean ± standard deviation or number (%) of patients.

a Percentage of ideal body weight.

b Percentage of weight change.

c (%lymphocytes × leukocytes)/100.

All patients had their anthropometric measures taken, except for the percentage of ideal body weight, which was not calculated for six subjects (35/41). None of these variables reached statistical significance (Table 2).

No preoperative biochemical variable, including serum albumin, reached statistical significance (Table 2). When analyzing albumin in the postoperative period, i.e., now influenced by the inflammatory response, its serum levels measured on the first (*p* = 0.021) and the seventh POD (*p* = 0.027) were the only variables that significantly differed between the two groups (Table 3).

**Table 3:** Serum albumin levels measured preoperatively and on day 1, day 3, day 5 and day 7 after surgery in both study groups.

	Complicated (n = 22)	Non-complicated (n = 19)	*p*-Value
Preoperative albumin	4.11 ± 0.38	4.24 ± 0.42	0.218
First-POD albumin	2.65 ± 0.49	3.04 ± 0.56	0.021
Third-POD albumin	2.85 ± 0.52	3.14 ± 0.51	0.07
Fifth-POD albumin	3.17 ± 0.58	3.40 ± 0.54	0.123
Seventh-POD albumin	3.21 ± 0.63	3.64 ± 0.57	0.027

Data are expressed as mean ± standard deviation.

In a logistic regression model after adjusting for confounders (age, T stage, clinical stage, previous radiotherapy, and presence of comorbidities), first-POD serum albumin remains correlated with complications (OR = 0.228; 95% CI [0.057–0.882]).

**Figure 1:**
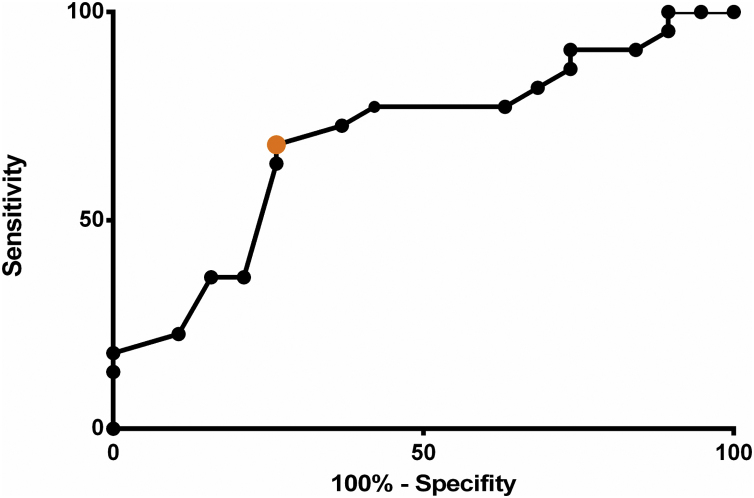
ROC curve showing sensitivity and specificity of different thresholds of postoperative serum albumin. Highlighted in orange is the cut-off value of 2.8 g/dL, the best threshold to distinguish between patients with and without postoperative complications.

The ROC curve was used to determine the accuracy and additional diagnostic properties of serum albumin measured on the first POD as a predictor of postoperative complications (Fig. 1). The overall discriminating power (accuracy), calculated from the area under the ROC curve, was 69.52% (95% CI 53.12%–89.64%; *p* < 0.001).

The diagnostic properties of serum albumin on the first POD are shown in Table 4.

**Table 4:** Sensitivity and specificity of different serum albumin levels measured on the first postoperative day in the diagnosis of postoperative complications.

	Sensitivity	Specificity	LR+^a^
<2.3 g/dL	22.73	89.47	2.15
<2.5 g/dL	36.36	84.21	2.30
<2.8 g/dL	68.18	73.68	2.59
<3.0 g/dL	77.27	57.89	1.83
<3.5 g/dL	90.91	15.79	1.08

a LR+, positive likelihood ratio.

**Figure 2:**
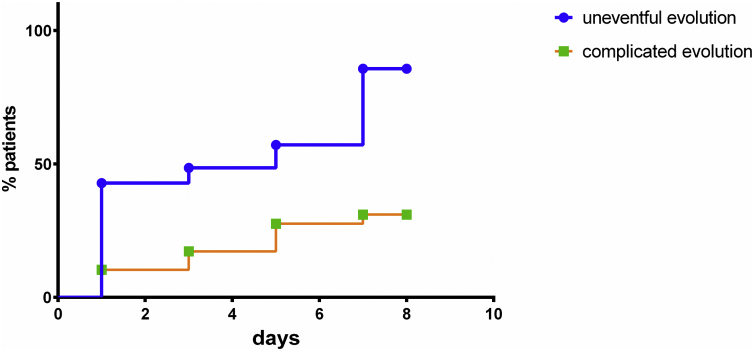
Time to albumin normalization curve for both complicated and noncomplicated groups.

The normalization of serum albumin levels occurred more frequently and significantly more rapidly in the group with no complications when compared to the complicated group (HR = 5.096; 95% CI: 1.973–13.16; *p* = 0.001) (Fig. 2).

## Discussion

Malnutrition is recognized to be a common issue among patients with head and neck cancer, with a prevalence in this population of 20%–67%.^1^, ^2^, ^3^ Methods of nutritional assessment should be reliable, practical, easily interpreted and inexpensive.^2^ All of them, however, have limitations, which is why a combination of variables is used in most cases. The lack of standardization in methods for diagnosing malnutrition leads to heterogenous study results, making it difficult to establish comparisons. Most of these studies focus only on variables such as weight loss, BMI, lean body mass loss or levels of serum proteins.^1^ In the present study, seven anthropometric variables and five biochemical ones, in addition to bioelectrical impedance, were used to determine which would be able to predict postoperative complications in patients with HNSCC.

The weight absolute value and its variation rate have prognostic properties.^12^ The %WC, calculated from the formula (AW-UW)/UW × 100,^13^ reflects the speed at which changes in body composition take place. By considering %WC and factoring time into the assessment, the severity of weight loss can be evaluated.^12^ The %IBW, on the other hand, is calculated from the formula (AW/IW) × 100 and is used to classify patients as having mild (80%–90%), moderate (70%–79%) or severe (<69%) malnutrition.^12^ Caution must be exercised when using this variable, as some healthy individuals weigh less than their ideal weight and in obese patients malnutrition may go unnoticed.

The prospective study conducted by Van Bokhorst-de van der Schueren et al. showed that a %WC greater than 10% over the past six months was the only nutritional variable associated with an increased risk of postoperative complications.^17^ However, a subsequent study by the same author evaluating survival rates found no significant correlation.^19^ While some authors have found patients with postoperative complications to have a greater reduction in body weight before^20^ or after surgery,^21^ others have found no correlation between these variables and postoperative morbidity and mortality.^3^, ^22^ In the present study, patients who had a decrease in %WC were not found to be at an increased risk of postoperative complications (*p* = 0.316), which was also the case for %IBW (*p* = 0.517), showing these variables were not able to predict postoperative complications in this patient population.

Studies assessing BMI in patients with HNSCC have produced conflicting results. A case-control study has shown these patients had a lower BMI than controls even before treatment.^23^ Some studies have found an association between low BMI and increased postoperative morbidity and mortality.^24^, ^25^ Others, on the other hand, have not found such a correlation.^21^, ^23^, ^26^ In the present study, mean BMI was 23.14 ± 3.73 kg/m^2^ and there was no significant correlation between low BMI and the risk of postoperative complications (*p* = 0.934). A possible explanation for this finding is that most study subjects were elderly patients, in whom height measurement is affected by vertebral compression, loss of muscle tone and postural changes.^27^ Despite that, the ease with which height can be measured and its correlation with morbidity and mortality found in some studies still justify using it in combination with other methods to assess nutritional status.

Very few studies have evaluated the role of skinfolds and limb circumferences in the assessment of HNSCC surgical patients. The present study evaluated the main skinfold and limb circumference variables in this population. There was no statistically significant difference in TSF (*p* = 0.811), AC (*p* = 0.269) or CC (*p* = 0.341) between patients with and without postoperative complications. Some limitations of TSF such as interindividual variation in body fat distribution, change in body composition due to age and nutritional status and age-dependent skinfold compressibility variation^28^ could partially explain why the present results differ from other findings in the literature. One study has shown patients with postoperative complications to have a greater reduction in TSF and AC after surgery,^21^ whereas a Brazilian study has shown patients with lower preoperative AC and TSF to have more postoperative complications.^22^

CC is the most sensitive measure for the assessment of muscle mass in the elderly,^21^ age group to which most patients with HNSCC belong. No study has nevertheless evaluated the role of CC in the diagnosis of malnutrition or as a predictor of postoperative complications in surgical HNSCC patients, making the present study a pioneer work in this regard.

Bioelectrical impedance analysis has been widely used in the past decades for nutritional assessment. Few studies have evaluated its role in the assessment of HNSCC surgical patients and none of them have found a correlation between BIA variables – LBM, BF and TBW – and nutritional status or postoperative morbidity in this population.^21^, ^23^, ^29^ The present study also did not found a statistically significant difference in preoperative TBW (*p* = 0.1341), LBM (*p* = 0.2167) or BF (*p* = 0.2051) between patients with and without postoperative complications.

Studies looking into hemoglobin levels and blood transfusion in HNSCC surgical patients have produced conflicting results. Retrospective studies^30^, ^31^ have found anemia, blood loss and perioperative blood transfusion to be risk factors for postoperative complications (infection and fistula). Prospective studies, however, have not found such a correlation.^21^, ^22^ In accordance with other prospective studies, the present study has also not found a difference regarding the need for blood transfusion between the groups with and without postoperative complications (*p* = 0.232). All patients had normal preoperative hemoglobin levels and the minor postoperative difference found between groups was not statistically significant (*p* = 0.891).

The ALC provides a rough estimate of immunological status.^10^ Different studies involving HNSCC surgical patients are in keeping with the present study in that they have found preoperative ALC to have no correlation with postoperative complications^17^, ^22^ or survival rates.^19^

Serum cholesterol usually decreases in late stages of malnutrition, limiting the use of this variable as a nutritional assessment tool. Nevertheless, some studies have been linking this reduction in cholesterol levels with increased morbidity and mortality^32^, ^33^ as well as with longer hospital stays.^33^

In the context of head and neck surgery, only one study investigated the potential association between preoperative hypocholesterolemia (serum cholesterol < 100 mg/dL) and the risk of pharyngocutaneous fistula,^34^ but found no correlation. In the present study, no patient had preoperative hypocholesterolemia and there was no statistically significant difference (*p* = 0.526) in mean total cholesterol between the groups with (191.9 ± 44.4 mg/dL) and without complications (182.9 ± 9.613 mg/dL).

The most commonly monitored hepatic protein in clinical practice is albumin.^35^ Inflammation elicited by trauma (surgical or otherwise) or acute disease (sepsis, pancreatitis) is the most important condition leading to decreased serum albumin levels. It does so directly, by stimulating transcapillary albumin extravasation from the intravascular to the extravascular space,^35^, ^36^ and indirectly, by promoting increased net protein loss following the onset of an inflammation-induced catabolic state and by inducing anorexia and thereby reducing nutrient intake – this phenomenon is called stress-induced hypoalbuminemia (SIH) and is not dependent upon the individual’s nutritional status.^36^

Many studies have looked into the relationship between serum albumin and morbidity and mortality rates among surgically treated patients with head and neck cancer, but only in the preoperative period.^17^, ^20^, ^21^, ^22^, ^26^, ^29^, ^31^^,^^34^ The analysis of the most important factor influencing the metabolism of hepatic proteins, i.e., the inflammatory response, has therefore been overlooked.

One study showed the difference between preoperative and first-POD serum albumin was able to predict major noninfectious complications in 59 cancer patients (*p* < 0.05).^37^ Only two head and neck surgery studies also evaluated postoperative albumin levels, but they produced conflicting results. The prospective study conducted by Liu et al.^24^ evaluated both preoperative and third-POD serum albumin levels and showed them to be both higher in the group without infectious complications. The prospective study conducted by Guo et al., on the other hand, found no statistically significant difference in either preoperative or postoperative serum albumin levels between groups (*p* > 0.80).^21^

In the present study, both preoperative and postoperative serum albumin levels were evaluated, thus taking into consideration their close relationship with the inflammatory response following surgical trauma. The association between time to normalization of serum albumin levels (“time to recovery”) and the risk of postoperative complications was also evaluated.

The data presented in this study showed first-POD serum albumin significantly differed between groups, thus establishing it as a predictor of complications following major head and neck surgery (*p* = 0.021). In addition, its overall discriminating power (accuracy) was calculated to be 69.52% (95% CI: 53.12–89.64%; *p* < 0.001) and a cut-off value of 2.8 g/dL was found to be the best threshold to distinguish patients with postoperative complications from patients with an uncomplicated postoperative course, yielding a sensitivity of 68.1% (95% CI: 45.13%–86.14%; *p* = 0.033) and a specificity of 73.68% (95% CI: 48.8%–90.85%; *p* = 0.033).

Hypoalbuminemia (serum albumin < 2.8 g/dL) on the first day after major head and neck surgery is associated with an increased risk of postoperative complications. The ability to predict such risk in an early manner would enable, for instance, differential nutritional supplementation strategies for at-risk patients or closer monitoring, e.g., keeping them in the hospital for longer periods.

In addition to occurring more often in patients with no complications, the normalization of serum albumin levels happened sooner in the noncomplicated group when compared to the complicated one (HR = 5.096; 95% CI: 1.973–13.16; *p* = 0.001). This phenomenon represents a normal physiological effect, indicates the improvement of the inflammatory response to surgical trauma and is associated with a lower risk of complications. The persistence of postoperative hypoalbuminemia despite adequate nutritional support indicates a higher risk of postoperative complications.

The serum albumin normalization curve evaluated the duration of SIH, which is directly associated with the stress caused by surgical trauma. In addition to monitoring postoperative albumin levels, it is important to know whether the patient’s serum levels normalized and how long it took them to do so, as the patients with no complications not only normalized their serum albumin levels but did so more often and five times more rapidly than the ones who developed complications. Those who managed to normalize their serum albumin levels and did so early on had consequently a lower risk of complications, even if they initially had postoperative hypoalbuminemia.

Seventh-POD serum albumin was also significantly different between groups (*p* = 0.027). However, as most complications following head and neck surgery occur in the first postoperative week, this variable would be a marker, and not a predictor, of postoperative complications. Moreover, all patients with hypoalbuminemia on the seventh POD already had it on the first POD.

The main limitation of this study was its sample size, which reduced the statistical power of some comparisons. The fact this study was conducted in a single institution weakens its external validity, as its findings may not reflect the actualities of other institutions. Lastly, not all patients in the sample had their body composition assessed by BIA and %IBW.

## Conclusion

Among the evaluated nutritional variables – bioelectrical impedance, anthropometry, and biochemical tests – serum albumin measured on the first postoperative day was significantly different between the two study groups, enabling it as a predictor of postoperative complications in the context of head and neck cancer. In addition, patients who managed to normalize their serum albumin levels and did so in an early manner, even with initial postoperative hypoalbuminemia, had a fivefold lower risk of complications.

## Funding

This research did not receive any specific grant from funding agencies in the public, commercial, or not-for-profit sectors.

## Conflicts of interest

The authors declare no conflicts of interest.
